# Alpha-Ketoglutarate: A Potential Inner Mitochondrial and Cytosolic Protector against Peroxynitrite and Peroxynitrite-Induced Nitration?

**DOI:** 10.3390/antiox10091501

**Published:** 2021-09-21

**Authors:** Joachim Greilberger, Michaela Greilberger, Reinhold Wintersteiger, Klaus Zangger, Ralf Herwig

**Affiliations:** 1 Otto Loewi Research Center for Vascular Biology, Immunology and Inflammation, Division of Physiological Chemistry, Medical University of Graz, 8010 Graz, Austria; 2 Schwarzl Medical Center, Institute of Scientific Laboratory, 8301 Graz, Austria; institut@laborwissenschaft.at; 3 Department of Pharmaceutical Chemistry, Institute of Pharmaceutical Sciences, University of Graz, 8010 Graz, Austria; reinhold.wintersteiger@uni-graz.at; 4 Institute of Chemistry, Organic and Bioorganic Chemistry, University of Graz, 8010 Graz, Austria; klaus.zangger@uni-graz.at; 5 German Medical Center, Department of Urology Surgery, Dubai 665664, United Arab Emirates; dr.ralf.herwig@gmail.com

**Keywords:** alpha-ketoglutarate (αKG), peroxynitrite (ONOO^−^), reactive oxygen and nitrogen species (RONS)

## Abstract

The generation of peroxynitrite (ONOO^−^) is associated with several diseases, including atherosclerosis, hypertension, neurodegeneration, cancer, inflammation, and sepsis. Alpha-ketoglutarate (αKG) is a known potential highly antioxidative agent for radical oxidative species such as peroxides. The question arises as to whether αKG is also a potential scavenger of ONOO^−^ and a potential protector against ONOO^−^-mediated nitration of proteins. NMR studies of 1 mM αKG in 100 mM phosphate-buffered saline at pH 7.4 and pH 6.0 were carried out in the presence or absence of a final concentration of 2 mM ONOO^−^. An ONOO^−^–luminol-induced chemiluminescence reaction was used to measure the scavenging function of several concentrations of αKG; quantification of αKG was performed via spectrophotometric enzymatic assay of αKG in the absence or presence of 0, 1, or 2 mM ONOO^−^. The nitration of tyrosine residues on proteins was measured on ONOO^−^-treated bovine serum albumin (BSA) in the presence or absence of 0–24 mM αKG by an ELISA technique using a specific anti-IgG against nitro-tyrosine. The addition of ONOO^−^ to αKG led to the formation of succinic acid and nitrite at pH 7.0, but not at pH 6.0, as αKG was stable against ONOO^−^. The absorbance of enzymatically estimated αKG at the time point of 30 min was significantly lower in favour of ONOO^−^ (1 mM: 0.21 ± 0.03, 2 mM: 0.12 ± 0.05 vs. 0 mM: 0.32 ± 0.02; *p* < 0.001). The luminol technique showed an inverse logarithmic correlation of the ONOO^−^ and αKG concentrations (*y* = −2 × 10^5^ ln(*x*) + 1 × 10^6^; *r*^2^ = 0.99). The usage of 4 mM αKG showed a significant reduction by nearly half in the chemiluminescence signal (284,456 ± 29,293 cps, *p* < 0.001) compared to the control (474,401 ± 18,259); for 20 and 200 mM αKG, there were further reductions to 163,546 ± 26,196 cps (*p* < 0.001) and 12,658 ± 1928 cps (*p* < 0.001). Nitrated tyrosine residues were estimated using the ELISA technique. A negative linear correlation was obtained by estimating nitrated tyrosine residues in the presence of αKG (*r*^2^ = 0.94): a reduction by half of nitrated tyrosine was estimated using 12 mM αKG compared to the control (326.1 ± 39.6 nmol vs. 844.5 ± 128.4 nmol; *p* < 0.001).

## 1. Introduction

αKG is widely known as an intermediate of the Krebs cycle and the natural ubiquitous collector of amino groups in body tissues. It has a potent ‘sparing’ effect on endogenous glutamine pools and a synergistic effect on the synthesis of glutamine. We have seen that αKG dramatically increases the synthesis of arginine, proline, and polyamines and reduces oxidative stress, which also play a key role in metabolic adaptation before and after surgery [1]. αKG is involved as a co-substrate in 2-oxo-glutarate-dependent dioxygenase and hypoxia inducing factor (HIF-1) and as a substrate of the Jumonji C domain-containing lysine demethylases (KDM2-7). Besides these functions, αKG is involved in the energy-generating process, wherein αKG is led by the formation of NAD^+^ over NADH^+^ to form carbon dioxide and succinyl-CoA or by an overflow of NADH^+^ to generate glutamate by up-regulating the glutamate dehydrogenase pathway. αKG can be formed enzymatically by the oxidative decarboxylation of isocitrate (isocitrate dehydrogenase), by the glutamate–pyruvate pathway (glutamate–pyruvate transaminase), and by the reversible transfer of amino groups from glutamate to oxalate by glutamate–oxaloacetate transaminase in either the inner-mitochondrial-membrane or cytoplasmic form.

αKG is known to react with H_2_O_2_ non-enzymically to form succinate and carbon dioxide in several biological systems, including cell cultures [2] in vitro and in vivo [3] and even in cell culture media alone [4]. We recently reported that αKG reduced oxidatively modified proteins in the presence of cigarette smoke radicals, estimated from the content of carbonyl proteins [5]. Additionally, our group reported that oral supplementation with αKG effectively increased the energy level and reduced the oxidative stress situation during surgery, as measured by the content of oxidatively modified proteins, compared to the placebo group, which resulted in a better recovery and lower hospitalisation time [1].

Peroxynitrite is a biological oxidising and nitrating agent generated physiologically by the superoxide anion radical (O_2_^•−^) and NO^•^ [6]. The nitration of tyrosine residues on proteins seems to take place in the normal ageing process [7], but also in the progress of a variety of diseases such as atherosclerosis, hypertension, neurodegeneration, inflammation, cancer, and sepsis [8,9]. For example, protein tyrosine nitration was shown to take place in the apoptosis of cultured motoneurons [10] and in an amyloid lateral sclerosis animal model during its progression [11].

So, strategies are needed to reduce peroxynitrite and the nitration levels of protein tyrosine. Indirect pathways such as increasing SOD activity by organic selenium compounds [12] or direct administration of endo- or exogenous uric acid resulted in a decrease of peroxynitrite and protein tyrosine nitration [13]. Inner-mitochondrial and cytoplasmic compounds such as αKG seem to have a positive effect in reducing peroxides, and it was speculated that αKG might also have antioxidative potential against peroxynitrite [14]. The aim of this study was to estimate the potential effect of αKG to reduce peroxynitrite and to inhibit protein tyrosine nitration.

## 2. Materials and Methods

### 2.1. Materials

The following materials Sigma Aldrich, Vienna, Austria, USA), NaNO_2_ (Sigma Aldrich, Vienna, Austria), NaOH (Merck, Darmstadt, Germany), MnO_2_ (Sigma Aldrich, Vienna, Austria), luminol (3-amino-phtalhydrazid, Sigma Aldrich, Vienna, Austria), bovine serum albumin (BSA; Sigma Aldrich, Vienna, Austria), rabbit anti-nitro-tyrosine IgG (Cayman Europe, Tallinn, Estonia USA), goat anti-rabbit IgG-HRP (Agilent Technologies, Santa Clara, USA), TMB and stop solution (Immundiagnostik, Bensheim, Germany), Tween 20 (Sigma Aldrich, Vienna, Austria), I-Block (ThermoFisher, Vienna, Austria), alpha-ketoglutarate (αKG), and an alpha-ketoglutarate enzymatic kit (Sigma Aldrich, Vienna, Austria).

### 2.2. Nuclear Magnetic Resonance Spectroscopy

Solutions of 1 mM αKG in 100 mM KPi at pH 7.4 and 6.0 were used. To enable field-frequency locking for the NMR experiments, 10% D_2_O was added. Peroxynitrite stock solutions (~20 mM) were added to yield final concentrations of 2 mM. All NMR spectra were acquired at 300 K on a Bruker Avance DRX 500 NMR spectrometer, equipped with a TXI 5 mm probe with z-axis gradients. Carbon chemical shifts were obtained from a 2D ^1^H-^13^C HSQC experiment.

### 2.3. Preparation of ONOO^−^

ONOO^−^ was prepared according to Hughes and Nicklin [15] by incipient mixing of equal volumes of 0.7 M H_2_O_2_ solution in 0.6 M HCl and 0.6 M NaNO_2_ on ice, followed immediately by termination of the reaction with 1.5 M NaOH. Surplus H_2_O_2_ was removed by the addition of a pinch of MnO_2_ and subsequent filtration of the suspension. The ONOO^–^ concentration was determined spectrophotometrically at 302 nm with an extinction coefficient of 1670 M^−1^ cm^−1^. Aliquots were stored at −70 °C until measurements.

### 2.4. Enzymatically Colorimetric αKG Quantification Assay Kit

In the assay, αKG was transaminated with the generation of pyruvate, which was utilised to convert a nearly colourless probe to colour (λ_max_ = 570 nm). The αKG standard was pre-incubated at room temperature for 30 min with different concentrations of ONOO^−^. Aliquots were transferred to a microtitration plate and mixed with assay buffer and reaction mix according to the protocol. After 30 min, the absorbance at 570 nm was measured. For kinetic experiments, OD 570 nm was measured every 5 min between 0 and 70 min.

### 2.5. ONOO^−^—Luminol Measurements Using αKG

The consumption of peroxynitrite was measured via a luminescence technique [16]. A volume of 5 µL of 30 mM ONOO^−^ was transferred to a white 96-well microtitration plate (Nunc, Roskilde, Denmark). Different concentrations of αKG were pipetted directly into the ONOO^−^. Immediately, after one second, luminol (400 µM 3-amino-phtalhydrazide in 10 mM PBS, pH 7.4) was added (total reaction volume 200 µL), and the chemiluminescence signal was detected on a BMG Lumistar plate reader (ServoLAB, Graz, Austria) each second for a duration of one minute. The luminescence signal was expressed in counts per second (cps).

### 2.6. Nitration of BSA with ONOO^−^

A quantity of 2 mg albumin was dissolved in 800 µL of 10 mM phosphate-buffered saline (pH 7.4) in the presence or absence of 0–24 mM αKG. Three volumes (400 µL) of ONOO^−^ solution were added in three steps (every 10 min) to obtain a total volume of 2 mL with an ONOO^−^ end concentration of 12 mM. The total reaction time was 30 min at 37 °C in a closed tube. After the reaction, a 1 mL aliquot was used immediately for the determination of nitro-tyrosine-BSA via an ELISA technique. One millilitre of nitrated tyrosine BSA was dialyzed without αKG in 10 mM PBS pH 7.4 overnight, changing the buffer solution 3 times (3 × 1 L). Dialyzed samples were spectrophotometrically (Beckmann spectrophotometer DU 640) measured at 438 nm, and the concentration of nitrated tyrosine on BSA was calculated with an extinction coefficient of e = 4300 M^−1^ cm^−1^ as described earlier. This nitrated tyrosine BSA was used as a standard for the ELISA technique.

### 2.7. Estimation of Nitrated Tyrosine BSA by an ELISA Technique

A quantity of 12 mM nitrated tyrosine BSA (2 mg/mL) was used as the standard. Volumes of 200 µL of several diluted BSA solutions (3.12, 1.56, 0.78, and 0.39 µg/mL) in the absence or presence of 12 mM αKG were applied to a transparent 96-well microtitration plate (Nunc, Roskilde, Denmark) and incubated at 37 °C for 2 h. After three washes (washing buffer: 10 mM PBS + 0.01 % tween 20, pH 7.4) with an Auto Plate Washer EL X 450 (BioTek, Bad-Friedrichshall, Germany), 250 µL of blocking solution (0.2% I-Block in 10 mM PNS, pH 7.4) was pipetted into the wells. After 30 min at room temperature (RT), the plate was washed again three times with washing buffer. A volume of 200 µL of 1:1000 diluted rabbit anti-nitrotyrosine IgG in 10 mM PBS, pH 7.4, was applied and incubated for 1 h at 4 °C. After a further washing step (3× *g*), 200 µL of goat anti-rabbit IgG-HRP (1:1000) was added to the samples and incubated for 1 h at RT. After at least six washes, 200 µL TMB solution was applied, and the reaction was stopped after 4 min at RT by adding 100 µL stop solution. Using a Power WaveX plate photometer (Bio-Tek, USA), extinctions of standards and samples were measured at 490 nm. The molar concentrations of nitro-tyrosine on BSA were calculated using the results of the photometrical measurements of 12 mM nitrated tyrosine BSA in the absence of any antioxidants (control). The standard curve was generated using the estimated absorbance. The results of all samples were expressed in nanomoles.

### 2.8. Statistical Analysis

Group comparisons were made using *t*-tests where appropriate and indicated. Linear regression and exponential regression curves were calculated based on Pearson regression (SPSS 25, SPSS Inc., Chicago, IL, USA). All values are given as the mean value and standard deviation. Statistical significance was considered at *p* < 0.01, with high significance at *p* < 0.001.

## 3. Results

### 3.1. Nuclear Magnetic Resonance Spectroscopy

**Figure 1:**
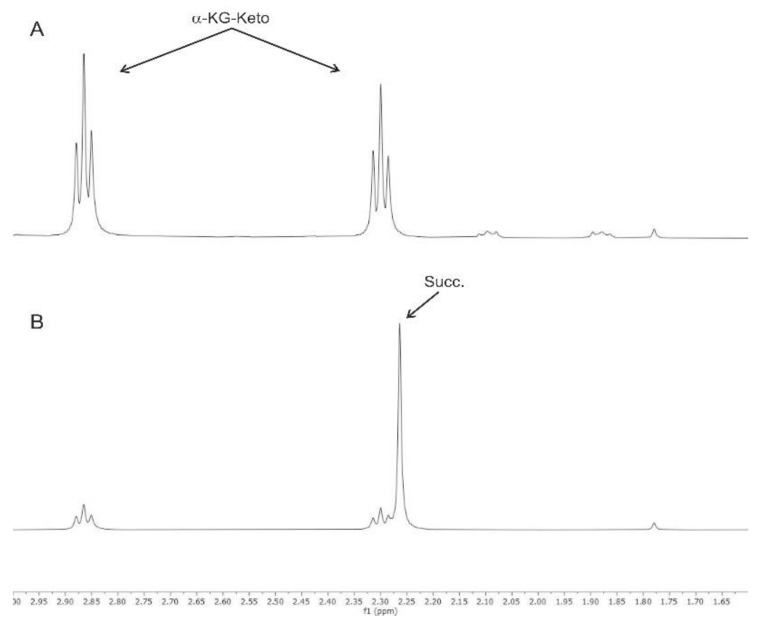
NMR spectra of 2-oxoglutarate (αKG) before and after the reaction with 2 mM ONOO− at pH 6.0 (**A**) and after the reaction with 2 mM peroxynitrite at pH 7.4 (**B**).

The α-KG-keto group of αKG is shown in Figure 1A; it gave the same signal after incubation with ONOO^−^ at pH 6.0, whereas the addition of ONOO^−^ to αKG led to the immediate formation of succinic acid at pH 7.4 (Figure 1B). The formation of succinic acid was confirmed by its carbon chemical shift as obtained through 2D ^1^H-^13^CHSQC.

### 3.2. Enzymatically Colorimetric Assay Kit for αKG Quantification

**Figure 2:**
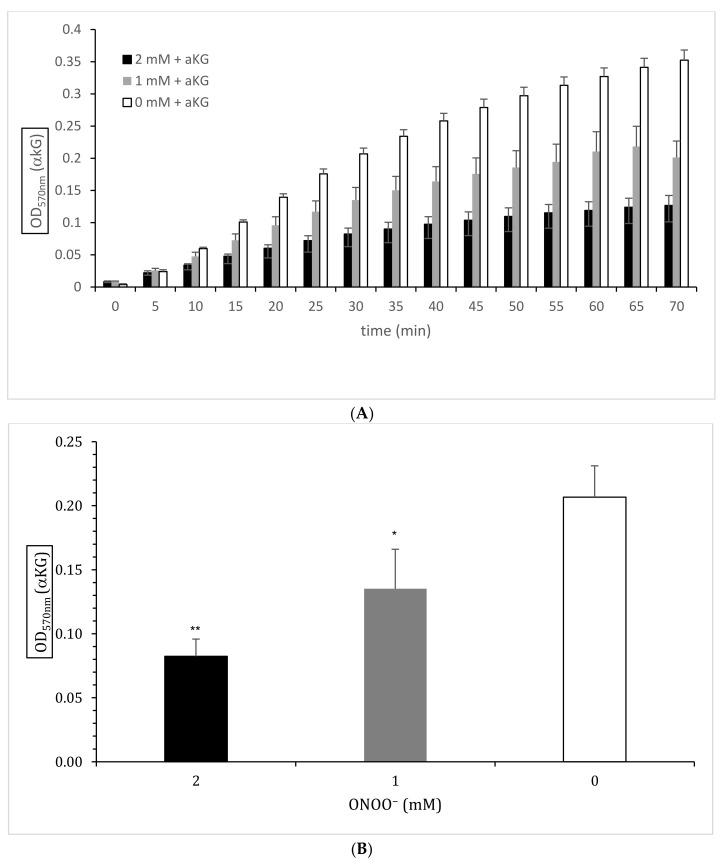
Spectrophotometric estimation of αKG (OD 570 nm) absorbance via an enzyme reaction mix in the absence or presence of 0 mM (white bars; *n*= 5), 1 mM (grey bars; *n* = 5), or 2 mM (black bars; *n* = 5) ONOO^−^ between 0 and 70 min (**A**) and after 30 min according to the protocol (**B**). * *p* < 0.01: significant compared to the control (0 mM ONOO^−^, white bar); ** *p* < 0.001: significant compared to the control (0 mM ONOO^−^, white bar).

Figure 2 shows the enzymatic estimation of αKG generated in the absence or presence of 0, 1, or 2 mM ONOO^−^ (*n* = 5). The kinetics of all three ONOO^−^ concentrations (0, 1, and 2 mM) between 10 and 70 min at OD 570 nm (A) showed a dose-dependent reduction of αKG by ONOO^−^ at each time point. The enzymatic reaction was not affected by ONOO^−^ and, therefore, was also not the active side of the enzyme. According to the protocol, Figure 2B shows the enzymatically estimated αKG concentrations after 30 min in the absence or presence of ONOO^−^ (*n* = 5). ONOO^−^ at 1 mM reduced the absorbance relating to the estimated αKG levels significantly compared to the control (0.207 ± 0.020 OD 570 nm vs. 0.135 ± 0.019 OD 570 nm; *p* < 0.01), but 2 mM ONOO^−^ reduced the absorbance even further (0.082 ± 0.009 OD 570 nm; *p* < 0.001).

### 3.3. ONOO^−^—Luminol-Induced Chemiluminescence in the Presence or Absence of αKG

**Figure 3:**
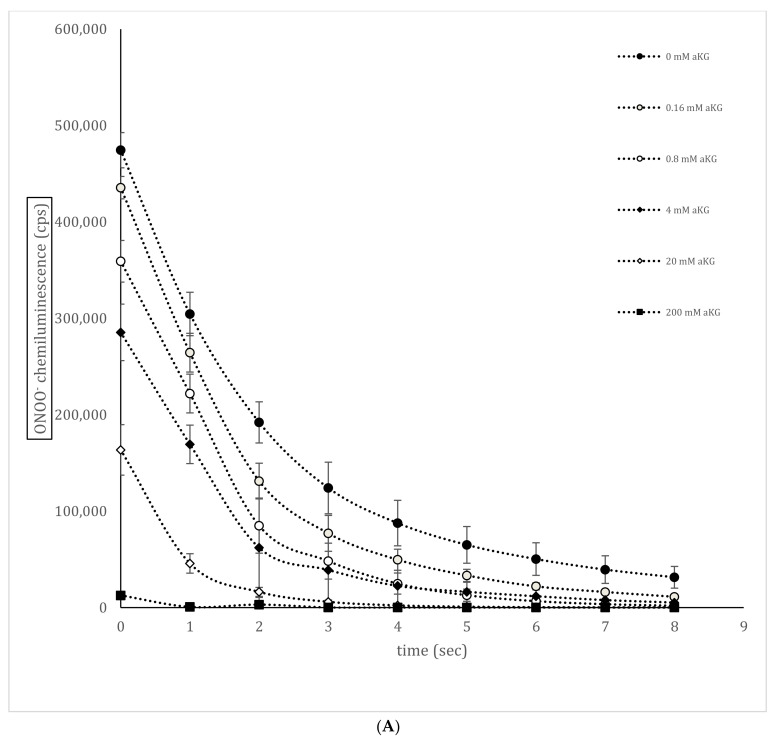

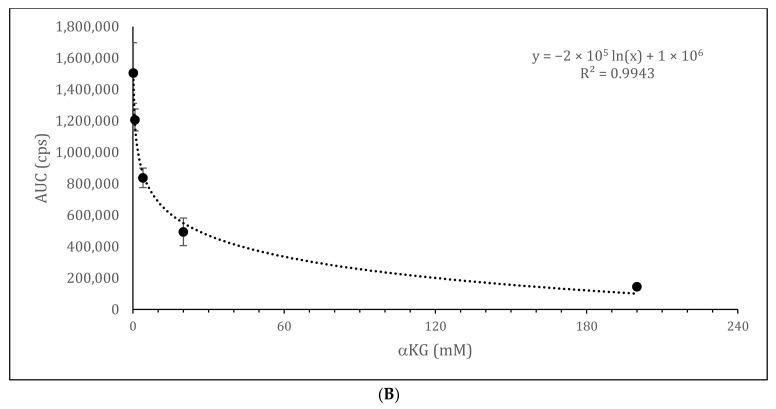
Standard curves of nitrated tyrosine residues on bovine serum albumin (BSA) using sandwich ELISA measurements (**A**; *n* = 5) and (**B**) calculated nitro-tyrosine residues on BSA protein in the absence or presence of 0–24 mM αKG. The relation and its regression term between nitrated tyrosine protein and 0 (control), 3, 6, 12, and 24 mM αKG concentrations (*n* = 5) were calculated. * *p* < 0.01: significant compared to the control; ** *p* < 0.001: significant compared to the control.

Figure 3A shows a representative measurement of the ONOO^−^–luminol-induced chemiluminescence curve in the presence or absence of different concentrations of αKG (*n* = 5). Using 0.8 mM αKG, a significant reduction in ONOO^−^ to 359,183 ± 21,604 cps was obtained compared to the control signal in the absence of any antioxidant (474,401 ± 18,259 cps; *p* < 0.001) at time point 0 s. Increasing the concentration to 4 mM αKG showed a reduction by nearly half (284,456 ± 29,293 cps; *p* < 0.001). Using 20 and 200 mM αKG further reduced the signal to 163,546 ± 26,196 cps (*p* < 0.001) and 12,658 ± 1928 cps (*p* < 0.001), respectively. The area under the curve (AUC; Figure 3B) of each curve was calculated and showed a negative logarithmic correlation to the αKG concentration, as shown in Figure 3B (*r* = 0.99; *y* = −2 × 10^5^ ln(*x*) + 1 × 10^6^). The calculated point of 50% reduction was around 12 mM αKG.

### 3.4. Nitrated Tyrosine Estimation on BSA in the Presence and Absence of αKG

**Figure 4:**
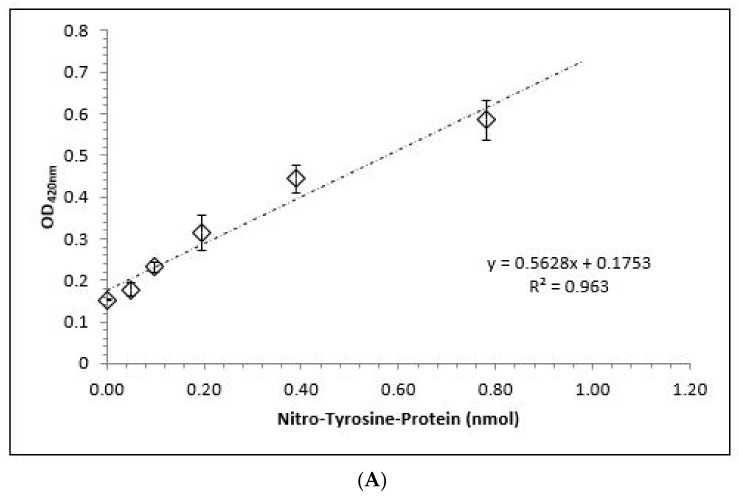

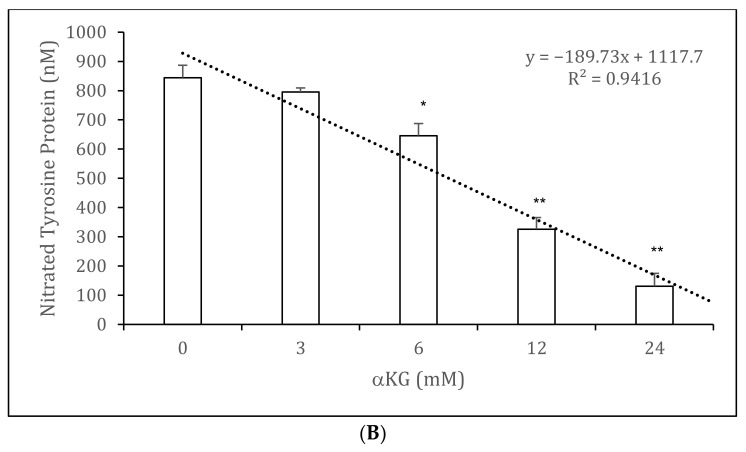
ELISA measurements of tyrosine residues on bovine serum albumin nitrated by ONOO^−^. (**A**) Standard curve of nitrated tyrosine protein (*n* = 5); (**B**) Estimation of nitro-tyrosine residues on bovine serum albumin in the absence or presence of 0 (control), 3, 6, 12, and 24 mM αKG (*n* = 5). The relation and its regression term between nitrated tyrosine proteins and αKG concentrations were calculated. * *p* < 0.01: significant compared to the control; ** *p* < 0.001: highly significant compared to the control.

Figure 4A shows the standard curve obtained by the ELISA technique with a highly linear correlation term of *r* = 0.94. Incubation of ONOO^−^ with BSA to generate nitro-tyrosine residues in the presence of 0–24 mM αKG led to a negative linear correlation with a high regression term of *r* = 0.97 (Figure 4B). While 3 mM αKG was not able to significantly decrease the amount of nitrated tyrosine residues on BSA compared to the control (795.5 ± 13.7 nM vs. 844.5 ± 42.3 nM), 6 mM was able to provide a significant reduction (646.1 ± 54.4; *p* < 0.01). Both 12 mM (326.1 ± 39.6 nM; *p* < 0.001) and 24 mM (130.6 ± 44.2 nM) αKG showed a highly significant reduction compared to the control.

## 4. Discussion

Recent years have witnessed an avalanche of new knowledge implicating free radicals in virtually every aspect of biology and medicine. It is now axiomatic that the regulated accumulation of RONS contributes to organismal health and well-being. RONS serve as signalling molecules involved in cell growth, differentiation, gene regulation, replicative senescence, and apoptosis [17].

Peroxynitrite is a strong oxidant that can be formed in vivo by the reaction of O_2_^•−^ and NO^•^. The discovery of peroxynitrite as a biological oxidant was seeded by combined data from the physiological and chemical literature [18]. Peroxynitrite is able to mediate oxidation and/or nitration in aqueous phases but also in hydrophobic milieux after free diffusion through membranes [9] to initiate lipid peroxidation and nitration, protein tyrosine nitration, and DNA modifications.

Antioxidant enzymes, such as superoxide dismutase (SOD), control the steady-state levels of peroxynitrite by reducing any overproduced O_2_^•−^, e.g., from macrophages, to H_2_O_2_. Uric acid, either endo- or exogenous, is a known antioxidative substance that scavenges peroxynitrite [14]. It is known that αKG is a potent antioxidative acting substance that reduces H_2_O_2_ to water and succinate. This compound is predominately formed in the inner mitochondria but is also formed quantitatively in cytoplasm. We showed for the first time that αKG is able to directly reduce peroxynitrite to succinate at physiological pH, as revealed by NMR. It appears that the affinity to reduce ONOO^−^ is very high because of the exponential decrease in the chemiluminescent signal. Using an enzymatic reaction in the quantification of αKG, ONOO^−^ was also able to eliminate αKG and not interfere with the enzymatic enzymes.

The nitration of tyrosine usually generates an additional negative charge and adds a relatively bulky substituent to the protein, which may affect the local charge distribution and/or conformation [9]. In vivo nitration of proteins in the presence of peroxynitrite is predominately estimated using the tyrosine residues of proteins such as bovine serum albumin (BSA), haem proteins, SOD, cytochrome c, or fibrinogen kinase glutathione S-transferase [19].

We used BSA as the example protein for the nitration of tyrosine residues because of its high level of tyrosine residues per protein. The nitration of tyrosine residues on BSA with ONOO^−^ was reduced by αKG: between 0 and 24 mM αKG showed a negative linear function in preventing the nitration of tyrosine residues. Combining these results with the αKG enzymatic reaction mix in the spectrophotometric assay, we speculate that the enzymatic active centre might be protected from ONOO^−^ by its substrate αKG. We suggest that this also takes place in other enzymes in which αKG is a co-factor in the inner mitochondrial membrane, as well as in the cytosol.

αKG is the obligate co-substrate of Fe(II)/2-oxoglutarate-dependent dioxygenases (OGDD), a superfamily of enzymes that catalyse the oxidative decarboxylation of αKG, producing succinate and CO_2_ from O_2_ [20]. Prolyl hydroxylation of hypoxia-inducible factor (HIF)-α, as catalysed by the Fe(II)/2-oxoglutarate (AKG)-dependent prolyl hydroxylase domain (PHD) enzymes, has a hypoxia-sensing role in animals [21].

Furthermore, the binding of prolyl-hydroxylated HIF-α to PHD2 is ∼50-fold hindered by prior αKG binding; thus, when αKG is limiting, HIF-α degradation might be inhibited by PHD binding [21]. Given that αKG is a limiting co-substrate for PHD activity during normoxia and that 2-oxoglutarate (αKG) levels depend on amino acid availability, it is possible that PHD activity depends not only on oxygen but also on amino acid availability. This suggests a global metabolic sensor function for PHDs, which could be signalling not only to HIF-α but also to mTOR [22]. We demonstrated first that αKG substitution has clear anticancer activity in vivo [23,24]. αKG was also able to reduce tumour growth and intra-tumoral perfusion [25,26]. Those findings were verified by another research group [27]. Furthermore, nitro-tyrosine residue levels on rat myocytes pretreated with an antioxidative solution containing αKG were significantly lower than those for control rats without pre-treatment [2].

αKG is a molecule involved in multiple metabolic and cellular pathways. Any loss of αKG, e.g., by peroxynitrite or hydrogen peroxide, might result in multiple dysfunctions due to αKG’s several functions as an energy donor, a precursor in amino acid biosynthesis, a signalling molecule, and a regulator of epigenetic processes and cellular signalling via protein binding. In vitro and in vivo antioxidative activities, protection against oxidative stress, and increased energy levels in αKG-supplemented humans were obtained in multiple studies [1,2,23].

αKG demonstrates a high potential to reduce peroxynitrite to body-suitable products such as succinate and nitrite, and it may protect against the nitration of mitochondrial and cytosolic proteins at neutral pH in cells and in blood. Nevertheless, further investigations are needed.

## Data Availability

The data presented in this study are available in the article.
